# Transfusion-related immunomodulation effect of erythrocyte characteristics on immune cells traits based on a multivariable Mendelian randomization study

**DOI:** 10.1097/JS9.0000000000004624

**Published:** 2026-01-19

**Authors:** Yujia Wang, Jinhuo Wang, Huan Wang, Zhanyu Cheng, Laiwei You, Bao-Ji Hu, Hao Li, Jie Wu, Lukun Wang, Yue Ju, Jianrong Guo

**Affiliations:** a Department of Anesthesiology, Shanghai Pudong New Area Gongli Hospital, Shanghai, 200315 China; b School of Gongli Hospital Medical Technology, University of Shanghai for Science and Technology, Shanghai, 200093, China; c Department of Anesthesia, Shanghai Pudong Hospital-Fudan University Pudong Medical Center, Shanghai, 201399 China

**Keywords:** erythrocyte characteristics, genome-wide association study (GWAS), hemoglobin, Mendelian randomization (MR), transfusion-related immunomodulation (TRIM)

## Abstract

**Background:**

Erythrocyte transfusion is the cornerstone of clinical practice, playing a vital role in treating various conditions such as severe blood loss, anemia, and certain hematological disorders. However, worries regarding the possible elevated hazard of transfusion-related immunomodulation (TRIM) persist.

**Purpose:**

We attempt to identify TRIM-associated erythrocyte characteristics and evaluate their genetic influence on TRIM based on a Mendelian randomization (MR) study.

**Method:**

A multivariate MR method was used to screen out the potential exposure factors. Gene variants related to the 12 erythrocyte characteristics were acquired from a genome-wide association study of hematologic characteristics, and these variants were utilized in the UK Biobank. Univariate MR methods were employed for sensitivity analyses. Hemoglobin was identified to be a significant erythrocyte trait in the context of TRIM. Based on the inverse variance-weighted (IVW) method, hemoglobin predicted by genetics was found to be favorably correlated with TRIM. MR-Egger, weighted median, and MR pleiotropy residual sum and outlier tests also provided robust estimation.

**Results:**

When incorporating all available gene variants for the erythrocyte characteristics, the exposure factor most associated with TRIM, based on marginal inclusion probability (MIP), was hemoglobin (MIP = 0.90), while all other erythrocyte characteristics had an MIP < 0.24. This indicates that hemoglobin stands out prominently among the various erythrocyte characteristics in its association with TRIM. The high MIP value for hemoglobin strongly suggests that it is a key determinant in the development of TRIM, while the much lower MIP values for other traits imply their relatively minor roles in this context. Sensitivity analyses showed that even when eliminating the highly correlated hematocrit, hemoglobin was still chosen with the greatest MIP. Additionally, univariate MR using IVW validated that hemoglobin was uniformly favorably correlated with TRIM.

**Conclusion:**

Hemoglobin is the key erythrocyte characteristic underlying TRIM, which shows that increasing hemoglobin via blood transfusion increases the risk of TRIM. Hence, the benefits of blood transfusion that raise hemoglobin need to be weighed against the side effects.

## Introduction

### Transfusion-related immunomodulation (TRIM)

Erythrocyte transfusion is frequently employed in oncological treatment, owing to tumor-associated anemia or therapy-related toxicity^[1]^. A recognized side effect of allogeneic erythrocyte transfusion is transfusion-related immunomodulation (TRIM), which gives rise to the modulation of the immune cells^[2,3]^. Erythrocyte concentrates comprise cell-based components, including remaining white blood cells and erythrocytes, and humoral substances, such as extracellular microvesicles, cytokines, and dissolvable human leukocyte antigen (HLA) peptides. Researchers debate all these factors as precipitating factors for TRIM, and they have not fully elucidated its fundamental mechanism. Extensive researches have been conducted to uncover the exact processes by which these factors interact to trigger TRIM. It is thought that the complex mixture of components in erythrocyte concentrates may initiate a series of molecular and cellular events. For example, the cytokines present might disrupt the normal signaling pathways within immune cells, while the extracellular microvesicles could transfer regulatory molecules between cells, contributing to the abnormal modulation of the immune cells. Understanding these mechanisms is crucial for developing strategies to mitigate the negative effects of TRIM during erythrocyte transfusions^[4]^.

### Mendelian randomization (MR)

Mendelian randomization (MR), which employs gene variants as instrumental factors, is less susceptible to confounding compared with conventional observational investigations and may aid in determining causative impacts^[2,5,6]^. The core principle of MR is based on the random assortment of genetic variants during gamete formation, which mimics the randomization process in randomized controlled trials (RCTs). By using genetic variants as instrumental factors, MR can isolate the causal associations between exposure factors and outcomes, reducing the influence of confounding factors that often plague traditional observational studies. This makes MR a powerful tool in medical research for uncovering true causal associations, especially when ethical or practical issues prevent the conduct of experimental studies. MR, at the intersection of experimental and observational research, furnishes a unique body of genetic proof regarding possible targets for interventions.

Multivariate MR simultaneously simulates multiple exposure factors, taking into account the measured pleiotropic effects through any of the observed exposure factors^[7,8]^. Previous research has utilized univariate and multivariate MR methods to evaluate the impacts of blood cell characteristics predicted by genetics on pathogenic risk^[9,10]^. Nevertheless, these methods have been restricted in statistical strength and their capacity to analyze high-dimensional, highly interrelated characteristics, like erythrocyte characteristics. To tackle these limitations, we utilized a new method for multivariate MR based on Bayesian model averaging (MR-BMA)^[11,12]^. Bayesian model averaging is a statistical approach that combines multiple models to account for model uncertainty. In the context of MR, MR-BMA can handle a large number of potential exposure factors simultaneously, which is crucial when dealing with high-throughput data such as those from genome-wide association studies (GWASs). It not only considers the individual effects of each exposure factor but also the correlations and interactions among them, providing a more comprehensive and accurate assessment of the associations among genetic variants, exposure factors, and outcomes. This method is applicable to high-throughput potential exposure factors and allows for the ranking of exposure factors within a Bayesian framework. MR-BMA functions effectively even when the exposure factors examined are highly interrelated due to biological mechanisms. This study strictly adheres to the guidelines for the use of artificial intelligence and does not use any artificial intelligence for manuscript writing^[13]^.

## Methods

### Study design

**Figure 1.**
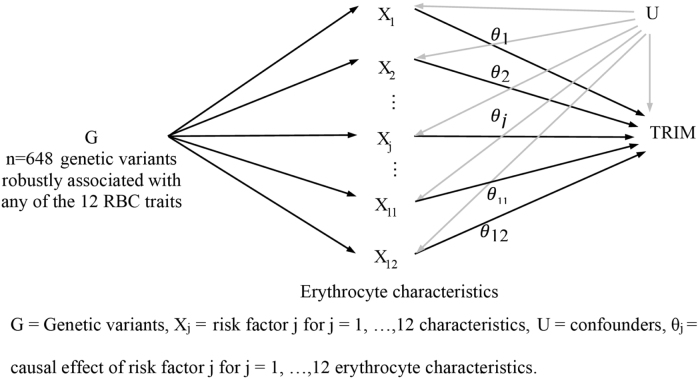
Major assumptions for a two-sample multivariable MR study.

This research is a two-sample multivariable MR study that depends on three instrumental factor assumptions (Fig. 1). First, the genetic variant is related to at least one of the exposure factors. Second, the genetic variant is independent of all confounding factors of each of the exposure–outcome associations. Third, the genetic variant is independent of the conditional outcomes on the exposure factors and confounding factors.

**HIGHLIGHTS**

- This study used multivariable Mendelian randomization with Bayesian model averaging to select and prioritize between 12 erythrocyte characteristics, which suggested endogenous hemoglobin is the key factor for transfusion-related immunomodulation (TRIM), with a detrimental effect in the general population.
- This study showed that increasing hemoglobin via blood transfusion increases the risk of TRIM.
- The benefits of therapies and other factors that raise hemoglobin need to be weighed against their risks.

### Genetic variants associated with erythrocyte characteristics

Genetic variants associated with erythrocyte characteristics were obtained from a current openly accessible GWAS with hematologic characteristics, which was performed in 173 480 individuals of European origin who had no hematological malignancy or other significant blood disorder^[9]^.

We acquired gene variants that strongly (*P* < 1 × 10^−9^) and independently (*r*^2^ < 0.001) predicted any of the 12 erythrocyte characteristics, including erythrocyte count, erythrocyte distribution width, reticulocyte fraction of erythrocytes, high light scatter reticulocyte percentage of erythrocytes, hemoglobin concentration, mean corpuscular hemoglobin (MCH), MCH concentration, mean corpuscular volume, hematocrit, reticulocyte count, immature fraction of reticulocytes, and high light scatter reticulocyte count. Genetic variants from the UK Biobank were assessed for imputation quality and validity as instrumental factors, using the following exclusion criteria: (1) an imputation information index <0.3 when the minor allele frequency (MAF) >3%, an information index <0.6 when the MAF is between 1 and 3%, an information index <0.8 when the MAF is between 0.5 and 1%, and an information index <0.9 when the MAF is between 0.1 and 0.5%; (2) deviation from Hardy–Weinberg equilibrium (HWE) at the Bonferroni-adjusted significance level; (3) being linked to possible confounding factors of the exposure–outcome causality at the Bonferroni-adjusted significance level.

### Genetic variants associated with transfusion-related immunomodulation

Genetic variants associated with TRIM were obtained from the GWAS Catalog (https://gwas.mrcieu.ac.uk/), derived from 3757 individuals of Sardinian origin^[14]^, consisting of both males and females, with an age span ranging from young adults to elderly individuals. Demographic characteristics of these participants were obtained as possible covariate factors, including age, gender, and body mass index, smoking habits, physical exercise, and health states. The comprehensive study covered 731 immune cell characteristics across 7 panels: T-B-NK cell, monocyte cell, myeloid cell, mature B cell, regulatory T-cell, classic dendritic cell, and T-cell maturation stages.

### Possible confounding factors

In the UK Biobank, to examine the randomization process, we evaluated the association of each genetic variant with possible confounding factors (i.e., identified risk factors that significantly affect both hematologic characteristics and the increased risk of TRIM). Variance analysis (for continuous variables) and chi-square tests (for categorical variables) were employed to evaluate whether each genetic variant was related to the possible confounding factors. The association of each genetic variant with TRIM was determined by an additive genetic model after adjusting for possible confounding factors.

### Multivariable Mendelian randomization based on Bayesian model averaging

Selection of exposure factors was carried out using MR-BMA^[15]^. Assuming that one of the considered models is true, MR-BMA orders all these submodels from the larger model in which all 12 erythrocyte characteristics could have a causative impact on TRIM (i.e., a single erythrocyte characteristic or a combination of multiple erythrocyte characteristics on TRIM), based on the posterior probability (PP) of their associations with the result. PP is obtained from a Bayesian model fitting criterion, which evaluates how effectively a linear combination of genetic associations with erythrocyte characteristics predicts the genetic associations with TRIM. The sum of the PP in each model is the marginal inclusion probability (MIP). Cook’s distance (*d* > the median variant of the relevant *F*-distribution) was used to identify influential variants. The *Q*-statistic (*Q* > 10) was used to identify outliers.

### Sensitivity analyses

Several univariate MR methods were utilized to confirm our findings from MR-BMA. An inverse variance-weighted (IVW) multiplicative random-effects meta-analysis of the genetic variant-specific Wald estimators was also employed. IVW offers uniform estimates only if all genetic variants are valid instrumental factors. As an extension of IVW, MR-Egger was used to detect horizontal pleiotropic effect only if the instrumental strength is independent of the direct impact. The MR-Egger intercept (*P* < 0.05) suggests the existence of a horizontal pleiotropic effect. Due to the limited statistical power of MR-Egger, the direction and effect size, rather than statistical significance, should be given more attention. The MR pleiotropy residual sum and outlier (MR-PRESSO) test was used to recognize and correct for horizontal pleiotropic outliers.

### Statistical analyses

MR-BMA was conducted following the code in GitHub (https://github.com/verena-zuber/demo_AMD). Univariable MR analyses were conducted using the R version 3.4.4 software with the TwoSampleMR and MR-PRESSO packages.

## Results

### Mendelian randomization based on Bayesian model averaging analyses

Based on exclusion criteria of imputation quality assessment, deviation from HWE, and association with possible confounding factors, 648 genetic variants were screened out from 731 genetic variants, which independently predicted any of the 12 erythrocyte characteristics at the genome-wide significance level. When incorporating all available genetic variants for the erythrocyte characteristics, the exposure factor most associated with TRIM was hemoglobin (MIP = 0.90); all other erythrocyte characteristics had an MIP < 0.24 (Table 1). To assess the model fit, we utilized the top-performing models with a PP > 0.02 (Table 2). Outlying variants (*n* = 7) were identified by their consistently high *Q*-statistics (*Q* > 10) in these top-performing models (Table 3). No influential variant was detected according to Cook’s distance (Supplemental Digital Content Table S1, available at: http://links.lww.com/JS9/G634).

**Table 1:** Ranking of erythrocyte characteristics according to their marginal inclusion probability (MIP).

	648 Genetic variants		641 Genetic variants	
Exposure	MIP	OR	MIP	OR
HGB	0.900	1.210	0.912	1.220
HCT	0.240	0.960	0.275	0.950
HLSR	0.180	0.980	0.154	0.980
RET%	0.110	0.990	0.108	1.010
MCHC	0.090	1.010	0.104	0.990
RBC	0.090	1.000	0.084	1.010
MCH	0.080	1.010	0.076	1.000
HLSR%	0.070	1.000	0.070	1.000
RET	0.070	1.000	0.067	1.000
IRF	0.070	1.010	0.060	1.010

HGB, hemoglobin concentration; HCT, hematocrit; HLSR, high light scatter reticulocyte count; RET%, reticulocyte fraction of red cells; MCHC, mean corpuscular hemoglobin concentration; MCH, mean corpuscular hemoglobin; HLSR%, high light scatter reticulocyte fraction of red cells; RET, reticulocyte count; IRF, immature fraction of reticulocytes.

**Table 2:** Ranking of models according to their posterior probability (PP).

	648 Genetic variants		641 Genetic variants	
Exposure	PP	OR	PP	OR
HGB	0.450	1.160	0.461	1.160
HCT, HGB	0.070	1.390	0.085	1.380
HGB, HLSR	0.040	0.940	0.034	0.940
HGB, RBC	0.030	0.940	0.024	0.940
HCT, HGB, HLSR	0.030	0.920	0.030	0.920
HGB, MCH	0.020	1.040	0.017	1.040
MCH, RBC	0.020	1.160	0.017	1.160
HCT, HGB, RET%	0.020	0.920	0.018	0.930
HGB, MCHC	0.020	1.070	0.019	1.070
HGB, HLSR, RET%	0.010	1.320	0.018	0.930

HGB, hemoglobin concentration; HCT, hematocrit; HLSR, high light scatter reticulocyte count; MCH, mean corpuscular hemoglobin; RET%, reticulocyte fraction of red cells; MCHC, mean corpuscular hemoglobin concentration.

**Table 3:** *Q* statistics for the best individual models and the maximum *Q* of each variant among these models for diagnostics.

SNP	Gene	*Q* M1	*Q* M2	*Q* M3	*Q* M4	*Q* M5	Max *Q*
rs77542162	ABCA6	20.79	20.82	20.43	20.34	20.36	20.82
rs174533	MYRF	16.28	16.31	15.94	15.72	15.88	16.31
rs11187938	TBC1D12	15.92	15.90	15.40	15.69	15.22	15.92
rs738408	PNPLA3	13.94	14.09	14.08	14.17	14.33	14.33
rs3747207	PNPLA3	13.49	13.67	13.63	13.73	13.91	13.91
rs139974673	CATSPER2P1	11.39	11.15	11.19	11.15	10.79	11.39
rs147233090	CATSPER2P1	11.00	10.76	10.77	10.77	10.39	11.00
rs78378222	TP53	7.99	8.29	8.37	8.44	8.89	8.89
rs11122449	GALNT2	8.74	8.58	8.71	8.75	8.48	8.75
rs41282676	EIF2AK1	7.49	7.44	8.42	7.45	8.63	8.63
rs2835349	AP000695.6	8.42	7.91	8.43	8.23	7.74	8.43
rs35979828	NFE2	8.08	8.34	7.90	7.68	8.20	8.34
rs9535495	DLEU7	7.71	7.83	7.04	7.69	7.00	7.83
rs17248895	PLEK2	7.29	7.36	7.53	7.20	7.69	7.69
rs1339847	TRIM58	7.65	7.34	5.16	7.69	4.19	7.69
rs4859682	SHROOM3	7.34	6.85	7.46	7.06	6.83	7.46
rs6880621	CTD-2197M16.1	6.71	7.12	6.65	6.83	7.19	7.19
rs972761	CTD-2197M16.1	6.45	6.83	6.39	6.55	6.90	6.90
rs6712203	COBLL1	6.44	6.73	5.99	6.43	6.22	6.73
rs4434553	TFR2	6.08	5.10	6.67	5.13	5.45	6.67
rs61750953	EGLN2	6.27	6.55	5.99	6.33	6.28	6.55
rs73652622	MIR4289	6.01	6.55	5.37	5.93	5.88	6.55
rs78415359	CTIF	6.16	6.27	6.17	6.37	6.33	6.37
rs833805	RP5-1120P11.1	5.74	6.32	5.51	5.99	6.20	6.32
rs72996113	RN7SL222P	5.67	6.12	5.01	5.56	5.39	6.12
rs13389219	COBLL1	5.82	6.07	5.40	5.79	5.61	6.07
rs964184	ZNF259	5.51	5.02	6.07	5.57	5.53	6.07
rs12548939	PVT1	5.89	5.73	5.99	5.51	5.79	5.99
rs1569419	PRDM16	5.97	5.68	5.99	5.55	5.60	5.99
rs56235845	RGS14	5.02	5.28	5.08	5.15	5.46	5.46

**Figure 2.**
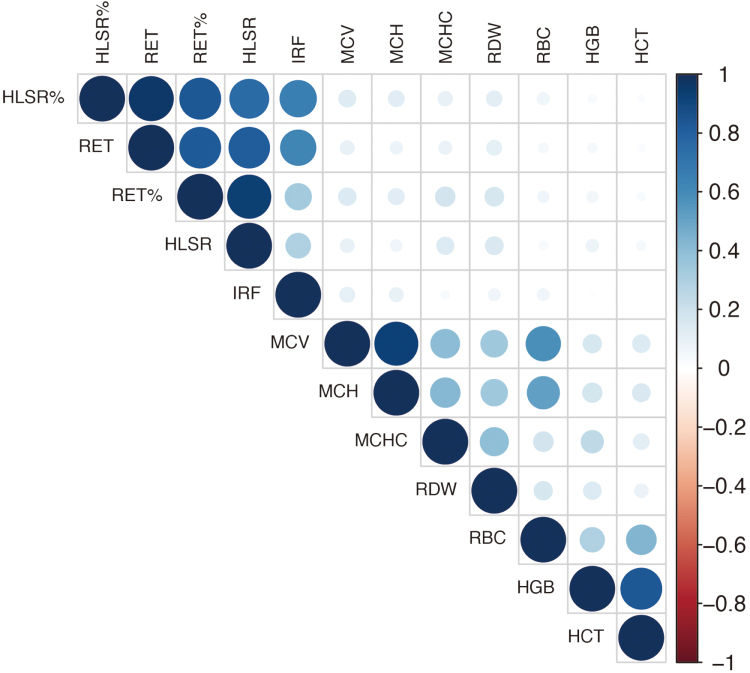
HGB, hemoglobin concentration; HCT, hematocrit; HLSR, high light scatter reticulocyte count; RET%, reticulocyte fraction of red cells; MCHC, mean corpuscular hemoglobin concentration; MCH, mean corpuscular hemoglobin; HLSR%, high light scatter reticulocyte fraction of red cells; RET, reticulocyte count; IRF, immature fraction of reticulocytes; MCV, mean corpuscular volume; RDW, red cell distribution width.

After excluding the seven outlying variants, we repeated the analyses. Hemoglobin (MIP = 0.91) was still the most relevant erythrocyte characteristic to TRIM, followed by hematocrit (MIP = 0.28) (Table 1). Notably, the genetic associations with hemoglobin and hematocrit were highly correlated (*r*^2^ = 0.91) (Fig. 2). When incorporating both hemoglobin and hematocrit, the models had a relatively low probability (PP = 0.085; Table 2). Five top-performing models were selected with a PP > 0.02, and no genetic variant with *Q* > 10 was identified (Supplemental Digital Content Tables S2 and S3, available at: http://links.lww.com/JS9/G634). Different initial PP parameters were used to test the robustness of the results, and the ranking of erythrocyte characteristics remained unchanged (Supplemental Digital Content Table S4, available at: http://links.lww.com/JS9/G634). As an additional sensitivity analysis, multiple sets of erythrocyte characteristics were used to repeat the analyses. Even when eliminating the highly correlated hematocrit, hemoglobin was still the most relevant erythrocyte characteristic to TRIM with the highest MIP. All these findings indicate that the impact of hemoglobin on TRIM is not significantly affected by the specific selection of erythrocyte characteristics.

### Univariable Mendelian randomization

The estimates for hemoglobin obtained through univariate MR are presented in Table 4, based on genetic variants after excluding those with known potential pleiotropic effects. Employing the IVW method, hemoglobin was consistently found to have a positive correlation with TRIM. The estimates from the weighted median, MR-Egger, and MR-PRESSO methods were of comparable magnitude and also showed consistent directional trends. This consistency strongly suggests that the influence of horizontal pleiotropy, which could introduce bias, is likely negligible in this context.

**Table 4:** Effect of genetically predicted hemoglobin concentration on the risk of TRIM using univariable MR.

Variant	Method	OR (95% CI)	*P* value	Intercept	*P* value^*^	*P* value^#^
81	IVW	1.21 (1.05–1.41)	0.01			
	MR-PRESSO	1.21 (1.06–1.38)	0.01			<0.0001
	MR-Egger	1.39 (1.04–1.87)	0.03	−0.006	0.3	
	Weighted median	1.18 (0.99–1.41)	0.06			
72	IVW	1.20 (1.05–1.37)	0.01			
	MR-PRESSO	No significant outliers				
	MR-Egger	1.28 (0.98–1.67)	0.08	−0.003	0.59	
	Weighted median	1.18 (1.00–1.40)	0.05			

*P value for MR-Egger intercept.^#^*P* value for global test, indicates horizontal pleiotropy

## Discussion

### Transfusion-related immunomodulation

**Figure 3.**
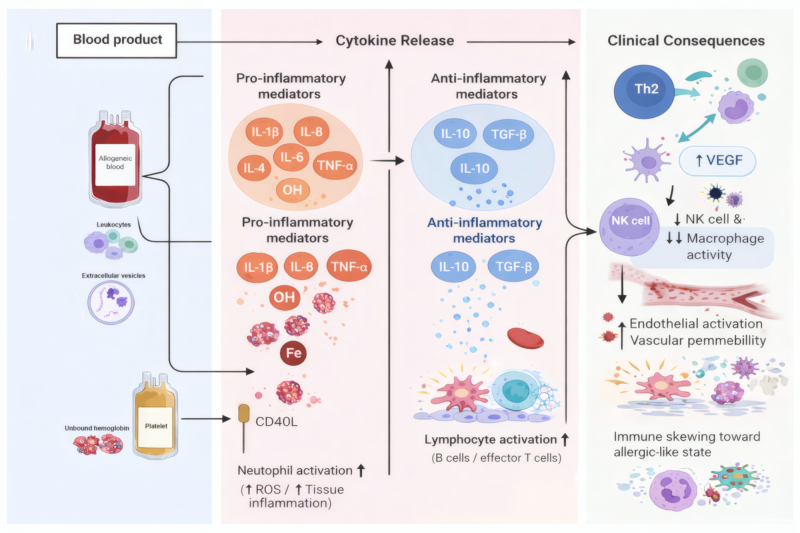
Mechanisms of transfusion-related immunomodulation (TRIM).

Numerous mechanisms elucidate how blood transfusions modify the immune system of the recipient (Fig. 3). Allogeneic blood products comprise numerous cellular and acellular components, such as leukocytes, extracellular vesicles (which contain microRNA, proteins, and lipids), unbound hemoglobin, and iron. Platelet-derived CD40 ligand has also been implicated during transfusions of platelets. These components release or initiate the release of pro-inflammatory and anti-inflammatory cytokines. These cytokines are able to evoke immune effects, including a Th2 cytokine response, a diminishment of NK cell and macrophage activity, and an augmentation in the activity of neutrophils, lymphocytes, and platelets. For instance, the pro-inflammatory mediators can activate immune cells, leading to the production of more cytokines and a cascade of immune reactions. The Th2 cytokine response, when overactivated, can shift the immune balance towards an allergic-like state, potentially affecting the body’s ability to fight off certain infections. The reduction in NK cell and macrophage activity weakens the body’s innate immune defenses, making the recipient more susceptible to pathogens. The augmented VEGF can promote angiogenesis, which might have implications for tumor growth in cancer patients, while the activation of endothelial cells can lead to changes in blood vessel permeability and the recruitment of immune cells to the site of inflammation.

Because some of these processes depend on leukocytes, leukoreduction has been conducted to alleviate these effects. In a prior study, when only patients who underwent transfusions were investigated, it was found that leukoreduction decreased the risk of postsurgical infection^[16]^. This finding implies that by reducing the proportion of leukocytes in transfused blood, the body’s risk of developing an infection after surgery can be significantly lowered, likely because there are fewer potential sources of immune-activating components or pathogens carried by these cells. Moreover, an RCT uncovered that the rates of HLA alloimmunization could be reduced through leukoreduction combined together with gamma irradiation^[17]^. Gamma irradiation, when used in conjunction with leukoreduction, may further inactivate or modify the components in the blood that trigger HLA alloimmunization, providing an additional layer of protection against immune reactions related to blood transfusions. However, another meta-analysis showed uncertain evidence as to whether leukoreduction could alter the risk of adverse reactions caused by blood transfusions^[18]^. The inconsistent results from different studies highlight the complexity of the effects of leukoreduction on transfusion-related outcomes, and more research is needed to fully understand its role and optimal application.

Despite the progress in surgical technology, cancer surgery remains among the surgeries with the greatest risk of blood transfusion^[19]^. Cancer surgery often involves significant blood loss due to the complex nature of cancerous tissue removal and the rich blood supply in these organs. The extensive tissue manipulation and potential damage to blood vessels during these procedures make blood transfusions a common necessity. In certain instances, blood transfusion is nearly unavoidable. Considering the relationship between perioperative blood transfusion and prognosis of tumor surgery, it is still crucial to assess whether blood transfusion affects cancer progression.

It has been discovered that patients undergoing hepatic resection and transplantation can reduce intraoperative allogeneic red blood cell transfusion without undermining oncological outcomes^[20]^. In hepatocellular carcinoma patients, intraoperative autologous blood transfusion has a relatively small impact on immune function in comparison with allogeneic blood transfusion^[21,22]^. The emerging evidence regarding the use of cell salvage may even boost immune function and survival in patients with renal cell carcinoma, prostate gland cancer, urinary bladder cancer, and cervical cancer^[23]^. However, in most clinical settings, the relationship between blood transfusion and clinical results is complicated. Interpreting observational studies is particularly challenging for numerous reasons. Anemia, including its severity, is separately linked to unfavorable outcomes during the perioperative period among most surgical patient groups^[24]^. Anemia can lead to reduced oxygen delivery to tissues, which may slow down wound healing, increase the risk of organ dysfunction, and weaken the overall immune response. Thus, it is challenging to distinguish the risk caused by anemia from the risk associated with red blood cell transfusion as a remedy for this condition. Additionally, anemia is more common among patients with complications that can influence long-term results, such as sepsis or organ malfunction. Sepsis can further disrupt the body’s normal physiological functions and immune response, and the presence of anemia may exacerbate these negative effects. Blood transfusion may lead to TRIM, which can increase the likelihood of tumor recurrence and other adverse outcomes related to blood transfusion^[1]^. TRIM can alter the body’s immune balance, potentially allowing cancer cells to evade the immune system and promoting tumor growth. Understanding these complex interrelationships is crucial for making informed decisions about red blood cell transfusion in clinical practice.

This MR research, which employs MR-BMA to select among 12 associated erythrocyte characteristics, indicates that hemoglobin is the erythrocyte characteristic most closely associated with TRIM. The primary strength of this research lies in the application of MR-BMA. It is used to identify and rank the potential drivers of TRIM among 12 erythrocyte characteristics, considering the extensive pleiotropy of highly associated erythrocyte characteristics. This is crucial because erythrocyte characteristics are often inter-related, and their pleiotropic effects can confound the identification of causal relationships. By accounting for these complex relationships, MR-BMA can more accurately pinpoint the traits that are truly associated with TRIM. Additionally, it offers verification and precise estimation of causal effects by means of univariate MR. Univariate MR provides a reliable way to cross-check the results obtained from MR-BMA, ensuring the validity and accuracy of the estimated causal effects.

However, several limitations of the current study should be recognized. First, although hemoglobin is associated with other erythrocyte characteristics, the results of our univariate MR are unlikely to be attributed to the pleiotropic effects of other erythrocyte characteristics. Second, MR-BMA does not offer impartial effect estimates. Due to the selection from a large number of genetic variants, the effect estimates of MR-BMA were reduced toward the null. The bias of the effect estimates is exchanged for reduced variance to strengthen and enhance the selection of genetic variants associated with erythrocyte characteristics. However, unbiased estimates were offered by univariate MR. Third, the GWAS of hematological traits applied high-quality procedures to optimize the precision of erythrocyte characteristics. Whereas, we cannot rule out the possibility that hemoglobin is more easily measured accurately than hematocrit. Fourth, our results reveal the average causal effects in the general population, which may not be applicable to all subgroups or be translated into the optimum level of hemoglobin in high-risk populations. Different subgroups within the population may have distinct genetic backgrounds, lifestyles, and underlying health conditions. For example, patients with certain genetic mutations or those suffering from specific diseases may respond differently to changes in hemoglobin levels. In high-risk populations such as cancer patients or the elderly, the optimal hemoglobin level may vary depending on factors like the stage of the disease, treatment regimens, and overall physiological status. Therefore, caution should be exercised when applying our findings to these specific subgroups.

## Conclusion

In conclusion, the present MR study suggests that hemoglobin is the key erythrocyte characteristic underlying TRIM, which shows that increasing hemoglobin via blood transfusion increases the risk of TRIM. Hence, the benefits of blood transfusion that raise hemoglobin need to be weighed against the side effect.

## Data Availability

The data that support the findings of this study are available from the corresponding author upon reasonable request. If necessary, we may also submit our experimental data in the form of attachments to journals for online publication.

## Research registration unique identifying number (UIN)

Not available.
